# Limited Impact of Pivalate-Induced Secondary Carnitine Deficiency on Hepatic Transcriptome and Hepatic and Plasma Metabolome in Nursery Pigs

**DOI:** 10.3390/metabo11090573

**Published:** 2021-08-25

**Authors:** Robert Ringseis, Sarah M. Grundmann, Sven Schuchardt, Erika Most, Klaus Eder

**Affiliations:** 1 Institute of Animal Nutrition and Nutrition Physiology, Justus-Liebig-University Giessen, Heinrich-Buff-Ring 26-32, 35392 Giessen, Germany; sarah.grundmann@ernaehrung.uni-giessen.de (S.M.G.); erika.most@ernaehrung.uni-giessen.de (E.M.); klaus.eder@ernaehrung.uni-giessen.de (K.E.); 2 Fraunhofer Institute for Toxicology and Experimental Medicine (ITEM), Nikolai-Fuchs-Str.1, 30625 Hannover, Germany; sven.schuchardt@item.fraunhofer.de

**Keywords:** carnitine deficiency, pivalate, liver transcriptome, plasma metabolome, pigs

## Abstract

Administration of pivalate has been demonstrated to be suitable for the induction of secondary carnitine deficiency (CD) in pigs, as model objects for humans. In order to comprehensively characterize the metabolic effects of secondary CD in the liver of pigs, the present study aimed to carry out comparative analysis of the hepatic transcriptome and hepatic and plasma metabolome of a total of 12 male 5-week-old pigs administered either pivalate (group PIV, *n* = 6) or vehicle (group CON, *n* = 6) for 28 days. Pigs of group PIV had approximately 40–60% lower concentrations of free carnitine and acetylcarnitine in plasma, liver and different skeletal muscles than pigs of group CON (*p* < 0.05). Transcript profiling of the liver revealed 140 differentially expressed genes (DEGs) between group PIV and group CON (fold change > 1.2 or <−1.2, *p*-value < 0.05). Biological process terms dealing with the innate immune response were found to be enriched with the DEGs (*p* < 0.05). Using a targeted metabolomics approach for the simultaneous quantification of 630 metabolites, 9 liver metabolites and 18 plasma metabolites were identified to be different between group PIV and group CON (*p* < 0.05). Considering the limited alterations of the hepatic transcriptome and of the liver and plasma metabolome, it can be concluded that pivalate-induced secondary CD is not associated with significant hepatic metabolism changes in pigs.

## 1. Introduction

Water-soluble carnitine is essential for the normal functioning of all tissues that contain mitochondria due to its well-documented role in the import of long-chain fatty acids into the mitochondrial matrix for subsequent β-oxidation [1]. Owing to its role in regulating the pool of free CoA, carnitine also affects the activity of enzymes involved in carbohydrate oxidation, such as pyruvate dehydrogenase [2], and thus, is generally important for energy metabolism. In line with this, carnitine deficiency (CD), which is defined by a plasma carnitine concentration below the physiological range (25–50 µmol/L in humans [3]), results not only in an impaired fatty acid utilization, but also in a decreased glucose tolerance and insulin sensitivity [4]. While primary CD, which is caused by mutations in the *SLC22A5* gene encoding for organic cation/carnitine transporter 2 (OCTN2) [5], is a relatively rare disease with strongly reduced (≈90%) plasma and tissue carnitine concentrations, secondary CD displays a less strong decrease (≈30–50%) of tissue carnitine concentrations, but is more frequent in humans. Individuals frequently affected by secondary CD are chronically ill patients suffering from cancer, chronic kidney disease, acquired immune deficiency syndrome, hepatitis C or hepatic encephalopathy [6,7,8,9]. In such patients, a decreased food intake, which is accompanied by a decreased intake of carnitine and specific micronutrients, such as iron, riboflavin and ascorbic acid, which are required as co-factors for carnitine synthesis, is one important reason for secondary CD. In addition, an insufficient carnitine synthesis and loss of carnitine during hemodialysis contributes to secondary CD in patients with chronic kidney disease. In cancer patients, moreover, treatment with chemotherapeutics accounts for secondary CD by reducing carnitine absorption and synthesis and/or increasing urinary carnitine excretion [10,11]. Sporadic cases of secondary CD are also known to occur as a result of long-term (>14 days) oral administration of pivalate-conjugated antibiotics, mainly in pediatric patients [12,13,14], because the pivaloyl moiety of these antibiotics binds free carnitine, leading to the formation of pivaloylcarnitine, which is excreted via urine in quantities exceeding endogenous carnitine synthesis.

Unlike primary CD, which exhibits a broad, but well-defined, clinical phenotype, such as hypoketotic hypoglycaemia, hepatic encephalopathy during infancy and early childhood, skeletal and cardiac myopathy and sudden death from cardiac arrhythmia [15], the more relevant secondary CD in humans is clinically less well defined and it is unclear which metabolic impairments result from secondary CD and which result from the underlying disease. In the rat, oral administration of pivalate was established as an experimental model to study the effects of isolated CD, in which free carnitine concentrations in the plasma, liver, heart and muscle are reduced by 20–40% [16,17,18]. Recently, we demonstrated that oral pivalate administration is also suitable for the induction of secondary CD in pigs, which are more appropriate model objects for humans than rats, as evidenced from an approximately 50% reduction in plasma and tissue (liver, skeletal muscle) carnitine concentrations [19]. Despite this marked decrease in tissue carnitine concentrations, biochemical and histological analysis revealed no changes of the metabolic and contractile phenotype of skeletal muscle in pivalate-treated pigs [19], indicating that secondary CD, in contrast to primary CD, is not accompanied by metabolic derangements or pathological alterations of skeletal muscle. Whether pivalate-induced CD causes metabolic alterations of the liver in pigs has not been investigated. In order to comprehensively characterize the metabolic effects of secondary CD in the liver of pigs, the present study aimed to carry out a comparative analysis of the hepatic transcriptome and the hepatic and plasma metabolome of pigs administered pivalate orally and control pigs administered vehicle only for 28 days using transcriptomics and targeted metabolomics. While transcriptomics makes it possible to unravel the molecular mechanisms underlying specific biological processes, targeted metabolomics enables the description of the biological alterations that occur in response to treatment.

## 2. Results

### 2.1. Growth Performance

Initial and final BW, daily BW gain and daily feed intake did not differ between group CON and group PIV (Initial BW: CON, 7.39 ± 1.16 kg; PIV, 7.63 ± 0.71 kg; final BW at day 28: CON, 18.0 ± 3.18 kg; PIV, 19.2 ± 2.73 kg; daily BW gain: CON, 0.38 ± 0.09 kg; PIV, 0.41 ± 0.07 kg; all *n* = 6/group; daily feed intake: CON, 0.66 ± 0.08; PIV, 0.67 ± 0.08; *n* = 3/group).

### 2.2. Plasma, Liver and Skeletal Muscle Carnitine Concentrations

**Figure 1:**
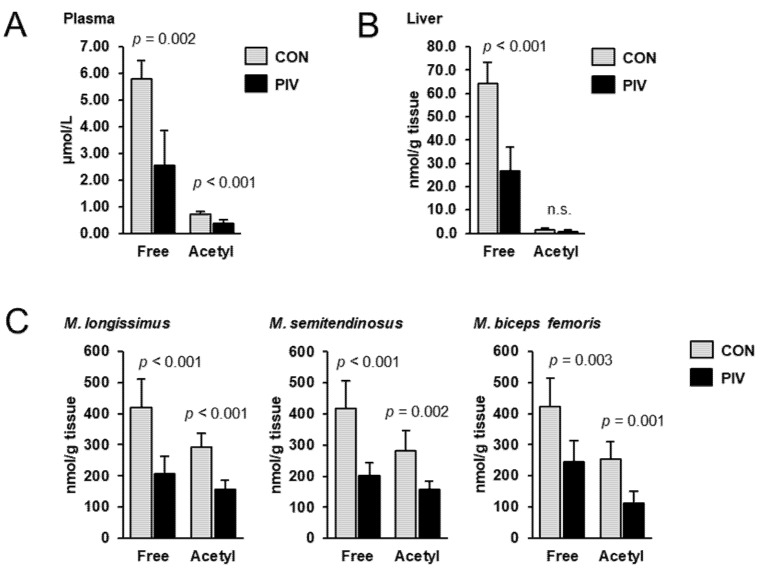
Concentrations of free carnitine and acetylcarnitine in plasma (**A**), liver (**B**) and different skeletal muscles (**C**) of 5-week-old pigs orally administered either vehicle (group CON) or pivalate (group PIV) for 28 days as determined by LC-MS/MS. Bars are means ± SD for *n* = 6 pigs/group.

To assess the carnitine status of the pigs, the concentrations of free carnitine and acetylcarnitine were determined in plasma, liver and different skeletal muscles using LC-MS/MS. In all tissues investigated, the concentration of free carnitine was higher than that of acetylcarnitine; regardless of treatment, the concentration of acetylcarnitine accounted for 12%, 2% and 60–70%, in plasma, liver and different skeletal muscles, respectively, of that of free carnitine (Figure 1). Concentrations of free carnitine in plasma, liver, *M. longissimus*, *M. semitendinosus* and *M. biceps femoris* were 56%, 59%, 51%, 52% and 42%, respectively, lower in group PIV than in group CON (*p* < 0.05). The concentrations of acetylcarnitine in plasma, *M. longissimus*, *M. semitendinosus* and *M. biceps femoris* were 47%, 46%, 44% and 56%, respectively, lower in group PIV than in group CON (*p* < 0.05). In the liver, the concentration of acetylcarnitine did not differ between the two groups.

### 2.3. Identification of Pivalate-Regulated Transcripts and of Biological Processes and Molecular Functions Affected by the Pivalate-Regulated Transcripts in the Liver

**Figure 2:**
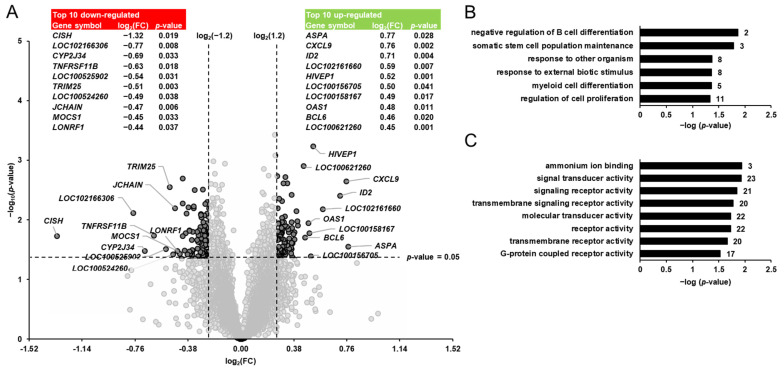
Identification of pivalate-regulated transcripts in the liver of pigs. (**A**): Volcano plot showing the differentially regulated hepatic transcripts between 5-week-old pigs orally administered either vehicle (group CON) or pivalate (group PIV) for 28 days. The double filtering criteria are indicated by horizontal (*p*-value < 0.05) and vertical (FC: > log_2_(1.2) or <log_2_(−1.2)) dashed lines. Transcripts in the upper left and the upper right corner represent the downregulated and the upregulated transcripts, respectively. The top 10 up- and downregulated transcripts with log_2_(FC) and *p*-value are shown in tabular form. (**B**,**C**): Enriched gene ontology (GO) biological process (**B**) and molecular function (**C**) terms assigned to the genes that were differentially regulated between group PIV and group CON. GO terms are sorted by their enrichment *p*-values (EASE score) (top: lowest *p*-value; bottom: highest *p*-value). The number of genes is shown next to the bars.

Differential transcript profiling was carried out to identify the pivalate-induced changes in the hepatic gene expression of the pigs. Considering the two filter criteria (FC > 1.2 or <−1.2; *p* < 0.05) a total of 140 transcripts out of 19,212 transcripts screened were identified as differentially expressed (upregulated: 76; downregulated: 64) in the liver of pigs between group PIV and group CON (Figure 2A). Amongst the upregulated genes, only three genes (*ASPA*, *CXCL9*, *ID2*) were regulated >1.5-fold. The top 10 upregulated transcripts were, in decreasing order of their FCs (in brackets), as follows: *ASPA* (1.70), *CXCL9* (1.69), *ID2* (1.64), olfactory receptor 7D2-like (*LOC102161660*; 1.50), *HIVEP1* (1.43), melanoma-associated antigen 8-like (*LOC100156705*; 1.42), olfactory receptor 5V1-like (*LOC100158167*; 1.41), *OAS1* (1.40), *BCL6* (1.38) and phosphatidylcholine transfer protein (*LOC100621260*; 1.37). Four genes (*CISH*, alpha-fetoprotein-like, *CYP2J34*, *TNFRSF11B*) amongst the downregulated genes exhibited a regulation that was <−1.5-fold. The top 10 downregulated transcripts were, in increasing order of their FCs (in brackets), as follows: *CISH* (−2.50), alpha-fetoprotein-like (*LOC102166306*; −1.71), *CYP2J34* (−1.61), *TNFRSF11B* (−1.54), phosphodiesterase 1C (*LOC100525902*; −1.45), *TRIM25* (−1.43), zinc finger protein 45 (*LOC100524260*; −1.40), *JCHAIN* (−1.39), *MOCS1* (−1.37) and *LONRF1* (−1.35). The FC and *p*-value of all differentially expressed transcripts between groups PIV vs. CON are shown in Supplementary Materials: Table S1.

To better interpret the biological meaning of the pivalate-induced changes in the hepatic transcriptome, GSEA was performed with the identified pivalate-regulated genes using GO biological process and molecular function terms. The GSEA of the transcripts that were differentially regulated between group PIV and group CON revealed that the enriched (*p* < 0.05) biological process terms were negative regulation of B cell differentiation, somatic stem cell population maintenance, response to other organisms, response to external biotic stimulus, myeloid cell differentiation, and regulation of cell proliferation (Figure 2B). The enriched (*p* < 0.05) molecular functions assigned to the transcripts that were differentially regulated between group PIV and group CON were ammonium ion binding, signal transducer activity, signaling receptor activity, transmembrane signaling receptor activity, receptor activity, molecular transducer activity, transmembrane receptor activity, and protein coupled receptor activity (Figure 2C).

### 2.4. Technical Validation of Microarray Data

Microarray data of 14 differentially expressed transcripts were validated by qPCR. As shown in Supplementary Materials: Table S2, the effect direction (positive or negative FC) was the same between microarray and qPCR for all validated transcripts, whereas the effect size (value of FC) differed to some extent for the validated transcripts between microarray and qPCR. Statistical analysis of qPCR data revealed that three (*ABHD5*, *HACL1*, *MOCS*) of the validated transcripts were regulated significantly (*p* < 0.05), whereas the other transcripts were not regulated (*p* > 0.05).

### 2.5. Identification of Pivalate-Regulated Metabolites in Plasma and Liver

**Figure 3:**
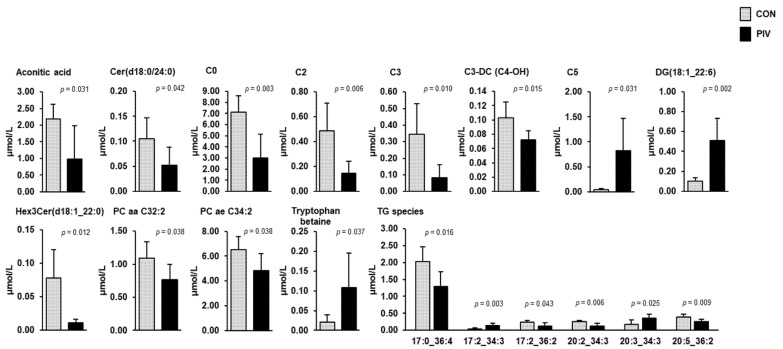
Concentrations of plasma metabolites regulated between 5-week-old pigs orally administered either vehicle (group CON) or pivalate (group PIV) for 28 days as identified by targeted plasma metabolomics. Bars are means ± SD for *n* = 6 pigs/group. Abbreviations: aa, acyl-acyl residue; ae, acyl-alkyl residue; C, carnitine; C0, free carnitine; C2, acetylcarnitine; C3, propionylcarnitine; C3-DC (C4-OH), malonylcarnitine (3-hydroxybutyrylcarnitine); C5, isovalerylcarnitine, valerylcarnitine, 2-methylbutyrylcarnitine and/or pivaloylcarnitine; DG, diacylglycerol; Cer, ceramide; PC, phosphatidylcholine; Hex3Cer, trihexosylceramide; TG, triacylglycerol species (with number of C-atoms and double bonds of one fatty acid and the sum of the number of C-atoms and double bonds of the other two fatty acids).

Targeted metabolome analysis of blood plasma and liver was performed to identify pivalate-induced changes in a large set of plasma and liver metabolites. In plasma, simultaneous quantification of 630 metabolites from different compound classes (acylcarnitines, alkaloids, amine oxides, amino acids, amino acid related, bile acids, biogenic amines, carbohydrates and related compounds, carboxylic acids, cholesteryl esters, ceramides, cresols, diglycerides, dihexosylceramides, dihydroceramides, fatty acids, hexosylceramides, hormones, indoles and derivatives, lysophosphatidylcholines, nucleobases and related compounds, phosphatidylcholines, sphingomyelins, triglycerides, trihexosylceramides, vitamins and their cofactors) revealed a total of 18 metabolites, whose concentrations differed between the two groups (Figure 3, *p* < 0.05). These metabolites belonged to the following classes: carnitine/acylcarnitines (C0, C2, C3, C3-DC (C4-OH), C5), amino acids-related (tryptophan betaine), carboxylic acids (aconitic acid), diacylglycerols (DG(18:1_22:6)), dihydroceramides (Cer(d18:0/24:0)), glycerophospholipids (PC aa C32:2, PC ae C34:2), glycosylceramides (Hex3Cer(d18:1_22:0) and triacylglycerols (TG(17:0_36:4), TG(17:2_34:3), TG(17:2_36:2), TG(20:2_34:3), TG(20:3_34:3), TG(20:5_36:2)). Amongst these metabolites, the concentrations of 13 metabolites (C0, C2, C3, C3-DC (C4-OH), aconitic acid, Cer(d18:0/24:0), PC aa C32:2, PC ae C34:2, Hex3Cer(d18:1_22:0), TG(17:0_36:4), TG(17:2_36:2), TG(20:2_34:3), and TG(20:5_36:2)) were lower and those of the remaining metabolites (C5, tryptophan betaine, DG(18:1_22:6), TG(17:2_34:3), and TG(20:3_34:3)) were higher in group PIV than in group CON (*p* < 0.05).

The sum of individual lipid species within the most important lipid classes, such as triacylglycerols, cholesteryl esters, phosphatidylcholine, lysophosphatidylcholine, sphingomyelin, diacylglycerols, glycosylceramides and ceramides, in plasma did not differ between group PIV and group CON (Table 1).

**Figure 4:**
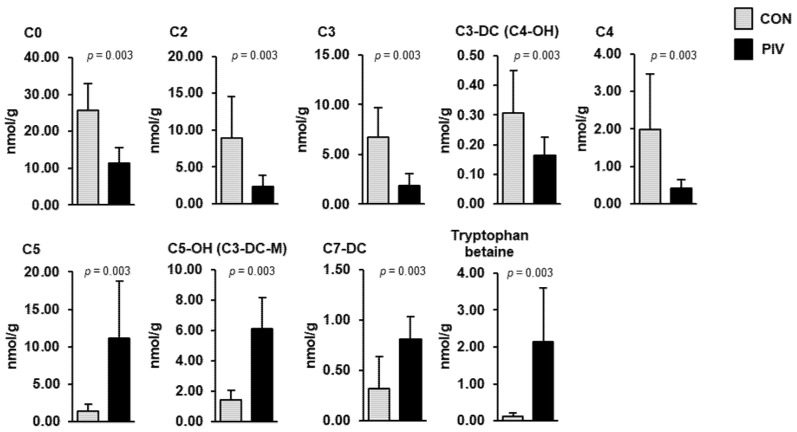
Concentrations of liver metabolites regulated between 5-week-old pigs orally administered either vehicle (group CON) or pivalate (group PIV) for 28 days as identified by targeted plasma metabolomics. Bars are means ± SD for *n* = 6 pigs/group. Abbreviations: C, carnitine; C0, free carnitine; C2, acetylcarnitine; C3, propionylcarnitine; C3-DC (C4-OH), malonylcarnitine (3-hydroxybutyrylcarnitine); C4, butyrylcarnitine; C5, isovalerylcarnitine, valerylcarnitine, 2-methylbutyrylcarnitine and/or pivaloylcarnitine; C5-OH (C3-DC-M), hydroxyvalerylcarnitine (methylmalonylcarnitine); C7-DC, pimelylcarnitine.

In the liver, the simultaneous quantification of 366 metabolites from different compound classes revealed nine metabolites that differed between groups PIV and CON (Figure 4, *p* < 0.05). Most of these liver metabolites belonged to the carnitine/acylcarnitines class (C0, C2, C3, C3-DC (C4-OH), C4, C5, C5-OH (C3-DC-M), C7-DC)) and one of these belonged to the amino acids-related class (tryptophan betaine). Hepatic concentrations of four metabolites (C5, C5-OH (C3-DC-M), C7-DC, tryptophan betaine) were higher in group PIV than in group CON (*p* < 0.05). The concentrations of five metabolites (C0, C2, C3, C3-DC (C4-OH), C4) in the liver were lower in group PIV than in group CON (*p* < 0.05).

## 3. Discussion

Pivalate-induced secondary CD occurs as an adverse effect of long-term treatment with pivalate-conjugated antibiotics, mainly in the pediatric population [12,13,14], because the absorbed pivaloyl moiety forms esters with free carnitine (pivaloylcarnitine), which are excreted via the urine, thereby causing carnitine depletion of tissues. In line with this, pigs of group PIV had approximately 40–60% lower concentrations of free carnitine and acetylcarnitine in the plasma and liver, and in different skeletal muscles, than pigs of group CON, as determined by LC-MS/MS. Reduced liver and plasma concentrations of free carnitine and various acylcarnitines in pigs of group PIV were also confirmed by liver and plasma metabolite profiling using a targeted metabolomics approach, according to which the concentrations of C0 (free carnitine), C2 (acetylcarnitine), C3 (propionylcarnitine), C3-DC (C4-OH) (malonylcarnitine and 3-hydroxybutyrylcarnitine) and C4 (butyrylcarnitine; only in the liver) were 58%, 70%, 76%, 29% and 79%, respectively, lower in group PIV than in group CON. In contrast, liver and plasma metabolite profiling revealed an 8- and 20-fold increase in the liver and plasma concentration, respectively, of the five-carbon acylcarnitine C5 in group PIV compared to group CON. The C5 signal in MS/MS analysis may represent four isomers, namely isovalerylcarnitine, valerylcarnitine, 2-methylbutyrylcarnitine and pivaloylcarnitine, because this analysis cannot differentiate C5-acylcarnitine isomers. It is highly likely that the marked elevation of plasma C5 levels in group PIV is due to elevated levels of pivaloylcarnitine as a consequence of pivalate administration. However, an increased urinary excretion of pivaloylcarnitine, which is known to occur in humans with secondary CD due to treatment with pivaloyloximethyl-esterified antibiotics [12], could not be shown in pigs of group PIV due to lack of urine collection in the present study. Nevertheless, our data strongly suggest that secondary CD was successfully induced in pigs by administration of pivalate.

In order to identify metabolic pathways and biological processes affected by secondary CD, genome-wide transcript profiling was performed in the liver of the pigs. Despite the pronounced depletion of carnitine levels in the liver, only 140 out of more than 19,000 genes screened were identified as differentially expressed according to the mild filter settings applied between group PIV and group CON. Apart from the low number of regulated transcripts, it is also noteworthy that the extent of regulation of these genes was very weak. To underline this, there was only one gene (*CISH*, cytokine inducible SH2-containing protein) that was regulated greater 2.0-fold between group PIV and group CON. In contrast, a recent transcriptomics study demonstrated that dietary supplementation of carnitine, which resulted in a 10-fold elevation of hepatic free carnitine concentration, regulates more than 500 hepatic genes greater 2.0-fold in growing pigs [20]. In line with the generally weak regulation of hepatic genes by pivalic acid treatment, qPCR validation of microarray data failed to confirm significant differences between groups for most of the validated genes despite the fact that the effect direction (up- or downregulation) could be confirmed for all genes by qPCR analysis. The failure of confirming the statistical significance level of microarray data in most of the validated genes is largely attributed to the small effect size (FCs of validated genes ranged between +1.70 and −1.43) in combination with the small group size (*n* = 6 animals) and the generally high variation of qPCR data, which generally complicates the possibility of confirming these microarray data. Thus, the microarray data from this study should not be overstated and must be interpreted very cautiously. Keeping these limitations in mind, bioinformatic GSEA of these 140 differentially expressed hepatic genes revealed a particular involvement of the encoded proteins in immune-related biological processes, such as regulation of B cell differentiation, myeloid cell differentiation, regulation of cell proliferation and response to external biotic stimulus. The pivalate-regulated genes responsible for the enrichment of these biological processes included *HIVEP1* (human immunodeficiency virus type I enhancer-binding protein 1), *ID2* (inhibitor of DNA binding 2), *BCL6* (B-cell lymphoma 6 transcription repressor)*, OAS1* (2’-5’-oligoadenylate synthetase 1), *CXCL9* (C-X-C motif chemokine ligand 9), *JUN* (Jun proto-oncogene), *IFIT3* (interferon-induced protein with tetratricopeptide repeats 3), *NR2E1* (nuclear receptor subfamily 2 group E member 1), *PRKCQ* (protein kinase C theta), *PPP1R16B* (protein phosphatase 1 regulatory subunit 16B), *SFRP1* (secreted frizzled-related protein 1), *TNFRSF11B* (TNF receptor superfamily member 11B), *IL12RB2* (interleukin 12 receptor subunit beta 2), *KLF1* (kruppel like factor 1), *FGF14* (fibroblast growth factor 14), *CISH*, *TRIM25* (tripartite motif containing 25), *TP53TG5* (TP53 target 5), *AFP* (alpha-fetoprotein), *ZFAND2A* (zinc finger AN1-type containing 2A), *LONRF1* (LON peptidase N-terminal domain and ring finger 1), *CYP2J34* (cytochrome P450 family 2 subfamily J member 34), *S100A4* (S100 calcium binding protein A4), and several others. Although these findings of a preferential involvement of the differentially regulated genes in immune-related biological processes might suggest that pivalate-induced CD causes a hepatic innate immune response at the molecular level, these findings should not be overstated due to the abovementioned limitations of our microarray analysis. 

In agreement with the weak changes of the hepatic transcriptome, targeted screening of hundreds of metabolites in the liver and plasma revealed only a low number (9 and 18, respectively) being different between pigs of group PIV and group CON. Apart from the above-mentioned carnitine/acylcarnitine species, the altered metabolites in plasma included mainly lipid species, such as triacylglycerol species, diacylglycerol species, phosphatidylcholine and lysophosphatidylcholine species, and ceramide and glycosylceramide species. In addition, tryptophan betaine, also called hypaphorine, in both liver and plasma, and aconitic acid in plasma, were identified as PIV-regulated metabolites. Despite the fact that most of the pivalate-induced changes in plasma were observed within lipid species, it has to be mentioned that all of them are only minor lipid species, which contribute only marginally to the total species within their lipid class. In agreement with this, the sum of individual lipid species within the dominant plasma lipid classes (triacylglycerols, cholesteryl esters, phosphatidylcholine, lysophosphatidylcholine, sphingomyelin, glycosylceramides, and ceramides) did not differ between groups. Considering this, it can be assumed that pivalate-induced CD is not associated with a profound effect on lipid metabolism in the liver. This assumption is supported by an earlier study in rats showing that treatment with pivalate does not cause metabolic changes in the liver of rats [21]. Obviously, this observation also applies to pigs. The indole alkaloid hypaphorine was identified in several legumes, including lentils, chickpeas and white beans [22], and was recently described as a suitable serum and urine biomarker of the intake of legumes [23]. Thus, the hypaphorine detected in both the liver and plasma of the pigs likely reflects the intake of soybean meal from the diet. Since pigs of both groups received identical diets, and feed intake was not different between groups, the higher liver and plasma concentration of hypaphorine in group PIV compared to group CON cannot be explained by a higher soybean meal intake. Despite being speculative, it is possible that the hepatic metabolism of this alkaloid was affected by pivalate treatment. Such an effect could be indicative of a confounding effect of pivalate administration rather than a consequence of secondary CD.

In conclusion, the present study shows that pivalate administration is an effective means for the induction of secondary CD in pigs, as shown by a 40–60% lowering of free and acetylcarnitine concentrations in plasma, liver and different skeletal muscles. The observation that pivalate-induced CD caused only weak alterations of the hepatic transcriptome and no profound changes of the liver and plasma metabolome indicates that pivalate-induced secondary CD is not associated with significant changes in hepatic metabolism in pigs.

## 4. Materials and Methods

### 4.1. Animal Experiment

The animal experiment was approved by the local Animal Care and Use Committee (Regierungspräsidium Giessen; permission no: GI 19/3 No. 30/2013). All experimental procedures described followed established guidelines for the care and handling of laboratory animals. For the experiment, a total of 12, 5-week-old, male crossbred piglets (Pietrain × (German Landrace × German Edelschwein)) were used, which were kept in pairs in flat-deck pens under controlled conditions (23 ± 2 °C room temperature, 50–60% relative humidity, light from 06.00 a.m. to 07.00 p.m.). The piglets were randomly allocated to two groups (group CON, group PIV) of *n* = 6 piglets each, and fed identical nutrient-adequate diets [24] according to a phase feeding system (phase I: until a body weight of 15 kg; phase II: until the end of the experiment) throughout the experimental period of 28 days. The diet in phase I consisted of (g/kg): wheat, 381.7; barley, 315; soybean meal (44% crude protein), 250; soybean oil, 15; mineral and vitamin premix, 33.5; L-lysin, 2.6; DL-methionine, 1.0; and L-threonine, 1.2. The diet of phase II consisted of (g/kg): wheat, 401.9; barley, 302; soybean meal (44% crude protein), 240; soybean oil, 15; mineral and vitamin premix, 33.4; L-lysin, 1.5; DL-methionine, 0.5; L-threonine, 0.7; and a mixture of organic acids (containing 45% formic acid, 7.7% lactic acid, 5% sodium acetate), 5. The carnitine concentration in the diets was below 10 mg/kg diet as analyzed by LC-MS/MS according to [25]. In addition, piglets were given orally either (per kg body weight (BW)) NaHCO_3_ (Sigma-Aldrich, Steinheim, Germany) solution (60 mg/100 mL) (group CON) or 30 mg sodium pivalate (95% purity; Sigma-Aldrich, Steinheim, Germany) dissolved in NaHCO_3_ solution (group PIV) each day throughout the experimental period of 28 days. Water was constantly available ad libitum from nipple drinkers during both experiments.

At the end of the experiment, the pigs were killed in the post-prandial period (2 h after their last meal) by electronarcosis followed by exsanguination. Blood was collected into heparinized polyethylene tubes (Sarstedt, Nümbrecht, Germany) and plasma was obtained by centrifugation at 1100× *g* for 10 min at 4 °C and subsequently stored at −80 °C. Aliquots from the liver and selected muscles (*longissimus dorsi*, *semitendinosus*, and superficial *biceps femoris*) were collected, snap-frozen in liquid nitrogen and stored at −80 °C pending analysis.

### 4.2. Carnitine Analysis in Plasma and Tissues

Liquid chromatography coupled with tandem mass spectrometry (LC-MS/MS) was used for determining the concentrations of free carnitine and acetylcarnitine in plasma, liver and different skeletal muscles according to [25]. Deuterated L-carnitine-methyl-d3-hydrochloride and deuterated O-acetyl-d3-carnitine-hydrochloride (both from Sigma-Aldrich, Steinheim, Germany) were used as internal standards.

### 4.3. Liver Transcriptomics

Total RNA from liver aliquots (20 mg) was extracted using TRIzol reagent (Invitrogen, Karlsruhe, Germany) according to the manufacturer’s protocol. RNA quantity and quality were determined spectrophotometrically and by electrophoresis, as described in detail in [26]. For microarray hybridization, RNA samples were processed at the Center of Excellence for Fluorescent Bioanalytics (Regensburg, Germany) using an Affymetrix GeneChip Porcine Gene 1.0 Sense Target array, which covers 19,212 genes represented by 394,580 probes, according to the manufacturer’s instructions. Following scanning of the processed GeneChips, cell intensity files, which provided a single intensity value for each probe cell, were generated from the image data using the Command Console software (Affymetrix). Correction of background and normalization of probe cell intensity data was carried out using the Robust Multichip Analysis (RMA) algorithm with the Expression Console software (Affymetrix). The RMA algorithm is a log scale multi-chip analysis approach fitting a robust linear model at the probe level to minimize the effect of probe-specific affinity differences. Expression levels of transcripts are measured using log transformed perfect match values, after carrying out a global background adjustment and across microarray normalization [27]. Annotation of microarrays was performed with the NetAffx annotation file “Porcine Annotations, CSV format, Release 36 (6.4 MB, 4/13/16)”. The microarray data of this study were provided in the NCBI’s Gene Expression Omnibus public repository (GSE178384) [28]. Owing to the rather moderate differences in the hepatic transcriptomes between group PIV and group CON, transcripts were defined as differentially expressed when the fold change (FC) between group PIV and group CON was >1.2 or <−1.2 and the *p*-value of the unpaired Student’s *t*-test was <0.05. Identical or similar filter criteria were also applied in several recent studies [26,29,30], in which treatment-induced changes of the transcriptome were only moderate and the application of more stringent filter criteria (e.g., false discovery rate and/or FC > 2.0 or <−2.0) failed to filter a sufficiently substantial number of genes to perform gene set enrichment analysis (GSEA). In the present study, filtering of differentially expressed transcripts using the Benjamini and Hochberg false discovery rate adjustment method could not be applied because the adjusted *p*-values for all transcripts were >0.05. GSEA was carried out with the list of differentially expressed transcripts in order to identify enriched Gene Ontology (GO) terms within the Biological process and Molecular function GO categories using the freely available DAVID 6.8 bioinformatic resource [31,32]. GO terms were considered as enriched if *p* < 0.05.

### 4.4. Validation of Microarray Data Using qPCR Analysis

Microarray data of 14 transcripts randomly selected out of the list of differentially regulated transcripts were validated by qPCR. Synthesis of cDNA and qPCR analysis was performed with a Rotor-Gene Q system (Qiagen, Hilden, Germany) using gene-specific primer pairs (Eurofins MWG Operon, Ebersberg, Germany) as described recently in detail [33]. Primer pairs were designed using Primer3 [34] and the Basic Local Alignment Search Tool [35]. Characteristics of primer pairs are shown in Supplementary Materials: Table S3. Normalization was carried out using multiple reference genes (*ACTB*, *RPS9*, *SDHA*) according to [36]. The mean value calculated from normalized individual values of group CON was set to 1 and the mean and SD of group PIV were scaled proportionally.

### 4.5. Liver and Plasma Metabolomics

Frozen liver tissue (200 mg) was homogenized in a mixture of ethanol/10 mM phosphate buffer (pH 7.5) (85/15, *v/v*) at a tissue:solvent ratio of 1:3 (*w/v*) using a TissueLyser (Qiagen, Hilden, Germany) along with tungsten carbide beads according to Zukunft et al. [37]. Since the extraction solvent used was appropriate to cover the polar metabolite classes of the metabolomics kit, only the polar metabolites, but not the unpolar metabolites (triacylglycerol and cholesterylester species), could be considered in the liver. After homogenization, the samples were centrifuged at 4 °C and 10,000× *g* for 5 min, and the supernatants were used for metabolite quantification. Quantification of plasma and extracted liver metabolites was carried out on an AB SCIEX 5500 QTrap™ mass spectrometer (AB SCIEX, Darmstadt, Germany) using the MxP™ Quant 500 kit (Life Sciences AG, Innsbruck, Austria), following the manufacturer’s protocols (https://biocrates.com/mxp-quant-500-kit; accessed at 15 December 2020). This targeted metabolomics approach allows for the simultaneous quantification of up to 630 metabolites from 26 compound classes. The extracts were analyzed by flow injection analysis tandem mass spectrometry (FIA-MS/MS) for lipids and liquid chromatography-tandem mass spectrometry (LC-MS/MS) using Agilent 1290 Infinity II liquid chromatography (Santa Clara, CA, USA) coupled with a tandem mass spectrometer for small molecules and using multiple reaction monitoring (MRM) to detect the analytes. Data were quantified using SCIEX Analyst^®^ software and imported into Biocrates MetIDQ™ software for the calculation of analyte concentrations, as well as data assessment and compilation.

### 4.6. Statistical Analysis

The statistical analysis was carried out using the SPSS 27 statistical software (IBM, Armonk, NY, USA). The experimental unit was the pen for daily feed intake and the individual animal for all other data. All parameters except liver transcriptomic data were tested for normal distribution using the Shapiro–Wilk test and tested for homoscedasticity with the Levene test. A Student’s *t*-test was performed for normally distributed and homoscedastic data. Normally distributed but heteroscedastic data were analyzed using the Welch t-test. If the normal distribution was not followed, a Mann–Whitney U test was performed. Pearson’s correlation coefficient (*r*) was used to evaluate the correlation between two variables. For all tests, differences were considered significant at *p* < 0.05.

## Figures and Tables

**Table 1:** Concentrations of important lipid classes in plasma of 5-week-old pigs orally administered either vehicle (group CON) or pivalate (group PIV) for 28 days as determined by targeted metabolomics.

Lipid Class	CON	PIV	*p*-Value
	µmol/L plasma		
Ceramides	1.69 ± 0.33	1.79 ± 0.39	0.610
Cholesterylesters	675 ± 106	685 ± 77	0.858
Diacylglycerols	9.09 ± 1.22	8.90 ± 2.62	0.875
Glycosylceramides	4.55 ± 0.69	4.63 ± 0.86	0.865
Lysophosphatidylcholine	95.2 ± 17.8	106.9 ± 15.9	0.257
Phosphatidylcholine	700 ± 139	704 ± 135	0.958
Sphingomyelin	71.9 ± 10.5	71.3 ± 8.1	0.910
Triacylglycerols	875 ± 195	832 ± 225	0.727

Data are means ± SD for *n* = 6 pigs/group.

## Data Availability

The microarray data were deposited in a MIAME-compliant format in the NCBI’s Gene Expression Omnibus public repository (GSE178384). The other datasets used and analyzed are available from the corresponding author on reasonable request.

## Supplementary Materials

The following are available online at https://www.mdpi.com/article/10.3390/metabo11090573/s1, Table S1: Fold change and *p*-value of all differentially expressed transcripts between group PIV and group CON, Table S2: qPCR validation of microarray data for selected differentially expressed transcripts, Table S3: Characteristics of gene-specific primers used for qPCR analysis.
