# Supplementary material for: Limited Impact of Pivalate-Induced Secondary Carnitine Deficiency on Hepatic Transcriptome and Hepatic and Plasma Metabolome in Nursery Pigs
Source: Metabolites. 2021 Aug 25;11(9):573. doi: 10.3390/metabo11090573 (PMC8468870; doi:10.3390/metabo11090573)
Supplement: Supplementary file 1 [file metabolites-11-00573-s001.zip › metabolites-1273832-supplementary.pdf]

Table S1 Fold change and *p*-value of all differentially expressed transcripts between group PIV and group CON

Gene symbol	Gene description	FC	*p*-value
Upregulated			
ASPA	aspartoacylase	1.70	0.0281
CXCL9	chemokine (C-X-C motif) ligand 9	1.69	0.0023
ID2	inhibitor of DNA binding 2, dominant negative helix-loop-helix protein	1.64	0.0039
LOC102161660	olfactory receptor 7D2-like	1.50	0.0067
HIVEP1	human immunodeficiency virus type I enhancer binding protein 1	1.43	0.0006
LOC100156705	melanoma-associated antigen 8-like	1.42	0.0411
LOC100158167	olfactory receptor 5V1-like	1.41	0.0166
OAS1	2-5-oligoadenylate synthetase 1, 40/46kDa	1.40	0.0114
BCL6	B-cell CLL/lymphoma 6	1.38	0.0197
LOC100621260	phosphatidylcholine transfer protein	1.37	0.0013
SPRY1	sprouty RTK signaling antagonist 1	1.33	0.0124
HTRA4	HtrA serine peptidase 4	1.32	0.0106
LOC100522693	putative olfactory receptor 56B2	1.32	0.0071
LOC102165149	olfactory receptor 51F2-like	1.31	0.0211
LOC100737604	FYVE, RhoGEF and PH domain-containing protein 6	1.31	0.0162
LOC100513557	olfactory receptor 51H1-like	1.30	0.0459
DACT2	dishevelled-binding antagonist of beta-catenin 2	1.30	0.0174
LOC106505929	lysine-specific demethylase 2B-like	1.29	0.0246
LOC100625836	heparan sulfate glucosamine 3-O-sulfotransferase 3A1	1.29	0.0415
AANAT	aralkylamine N-acetyltransferase	1.29	0.0138
GIPC2	GIPC PDZ domain containing family, member 2	1.29	0.0244
PLK3	polo-like kinase 3	1.29	0.0308
LOC100627486	olfactory receptor 4P4-like	1.29	0.0108
ADGRG2	adhesion G protein-coupled receptor G2	1.29	0.0038
MIR221	microRNA mir-221	1.28	0.0362
RND1	Rho family GTPase 1	1.28	0.0292

PQLC2	PQ loop repeat containing 2	1.27	0.0304
LOC106508264	olfactory receptor 52M1-like	1.27	0.0206
SLC13A1	solute carrier family 13 (sodium/sulfate symporter), member 1	1.27	0.0161
LOC100152003	olfactory receptor 2S2-like	1.26	0.0145
LOC100737856	olfactory receptor 51G1	1.26	0.0268
IQSEC2	IQ motif and Sec7 domain 2	1.26	0.0024
JUN	jun proto-oncogene	1.25	0.0128
CHAT	choline O-acetyltransferase	1.25	0.0019
CCDC82	coiled-coil domain containing 82	1.25	0.0238
LOC100513607	tripartite motif-containing protein 60-like	1.25	0.0339
PRKCQ	protein kinase C, theta	1.25	0.0381
LOC100737900	olfactory receptor 51G2	1.25	0.0487
SOX21	SRY (sex determining region Y)-box 21	1.24	0.0125
GPR18	G protein-coupled receptor 18	1.24	0.0088
IL12RB2	interleukin 12 receptor, beta 2	1.24	0.0351
LOC100156296	olfactory receptor 6C6	1.24	0.0240
LOC100514119	olfactory receptor 52R1	1.24	0.0108
BCL9L	B-cell CLL/lymphoma 9-like	1.24	0.0275
SH2D2A	SH2 domain containing 2A	1.23	0.0024
CHRNA9	cholinergic receptor, nicotinic, alpha 9 (neuronal)	1.23	0.0213
TMPRSS4	transmembrane protease, serine 4	1.23	0.0344
C9H11orf71	chromosome 9 open reading frame, human C11orf71	1.23	0.0280
CYLC1	cylicin, basic protein of sperm head cytoskeleton 1	1.23	0.0143
ADGRV1	adhesion G protein-coupled receptor V1	1.23	0.0092
IFIT3	interferon-induced protein with tetratricopeptide repeats 3	1.23	0.0165
NR2E1	nuclear receptor subfamily 2, group E, member 1	1.23	0.0049
TACR2	tachykinin receptor 2	1.23	0.0110
SEPTIN6	septin 6	1.22	0.0136
LOC100512910	olfactory receptor 10G3	1.22	0.0171
DUSP2	dual specificity phosphatase 2	1.22	0.0207
KLHL33	kelch-like family member 33	1.22	0.0368
LRRC18	leucine rich repeat containing 18	1.22	0.0463

TMEM178A	transmembrane protein 178A	1.22	0.0389
FGF14	fibroblast growth factor 14	1.22	0.0317
TMEM163	transmembrane protein 163	1.22	0.0175
GPX2	glutathione peroxidase 2	1.21	0.0181
KLF1	Kruppel-like factor 1 (erythroid)	1.21	0.0376
MIR551A	microRNA mir-551a	1.21	0.0190
KEL	Kell blood group, metallo-endopeptidase	1.21	0.0444
ZAP70	zeta-chain (TCR) associated protein kinase 70kDa	1.21	0.0492
LOC106504774	centromere protein J-like	1.21	0.0333
KLF3	Kruppel-like factor 3 (basic)	1.21	0.0307
PPP1R16B	protein phosphatase 1, regulatory subunit 16B	1.21	0.0442
LOC100514488	olfactory receptor 3A2	1.21	0.0388
LOC106506271	sodium channel protein type 1 subunit alpha-like	1.21	0.0208
DNAJC5B	DnaJ (Hsp40) homolog, subfamily C, member 5 beta	1.21	0.0044
ZDHHC15	zinc finger, DHHC-type containing 15	1.21	0.0394
GNAT3	guanine nucleotide binding protein, alpha transducing 3	1.21	0.0116
TOX	thymocyte selection-associated high mobility group box	1.21	0.0284
GPR52	G protein-coupled receptor 52	1.21	0.0113
Downregulated			
TRAPPC2	trafficking protein particle complex 2	-1.21	0.0476
LOC102165624	olfactory receptor 2AE1-like	-1.21	0.0150
BASP1	brain abundant, membrane attached signal protein 1	-1.21	0.0368
SLC25A47	solute carrier family 25, member 47	-1.21	0.0397
CRELD2	cysteine-rich with EGF-like domains 2	-1.21	0.0396
OTUB2	OTU deubiquitinase, ubiquitin aldehyde binding 2	-1.21	0.0469
C13H3orf17	chromosome 13 open reading frame, human C3orf17	-1.21	0.0031
UNC119B	unc-119 lipid binding chaperone B	-1.21	0.0293
SLA-7	MHC class I antigen 7	-1.21	0.0321
AEN	apoptosis enhancing nuclease	-1.21	0.0355
NDFIP2	Nedd4 family interacting protein 2	-1.22	0.0091
LOC100522040	glutamate formimidoyltransferase-like	-1.22	0.0249

LOC100518088	uncharacterized LOC100518088	-1.22	0.0079
PIGH	phosphatidylinositol glycan anchor biosynthesis, class H	-1.22	0.0207
GMPPB	GDP-mannose pyrophosphorylase B	-1.22	0.0083
POGK	pogo transposable element with KRAB domain	-1.22	0.0345
TOR1A	torsin family 1, member A (torsin A)	-1.22	0.0131
NDUFB5	NADH dehydrogenase (ubiquinone) 1 beta subcomplex, 5, 16kDa	-1.22	0.0177
LOC100518801	pro-opiomelanocortin-like	-1.23	0.0239
LOC102165772	uncharacterized LOC102165772	-1.23	0.0150
LOC100154839	tumor necrosis factor receptor superfamily member 10A-like	-1.24	0.0181
FREM1	FRAS1 related extracellular matrix 1	-1.24	0.0406
CBX4	chromobox homolog 4	-1.24	0.0235
PRDM13	PR domain containing 13	-1.24	0.0408
LOC102158532	putative ATP-dependent RNA helicase DHX57	-1.24	0.0305
SLC22A5	solute carrier family 22 (organic cation/carnitine transporter), member 5	-1.24	0.0218
LOC102162630	putative protein ZNF720	-1.25	0.0159
CRISP3	cysteine-rich secretory protein 3	-1.25	0.0487
S100A4	S100 calcium binding protein A4	-1.26	0.0445
PHPT1	phosphohistidine phosphatase 1	-1.26	0.0031
LOC102159828	40S ribosomal protein S20 pseudogene	-1.26	0.0064
LOC100623627	zinc finger protein 583	-1.26	0.0059
ORC1	origin recognition complex, subunit 1	-1.27	0.0158
LOC100523301	olfactory receptor 1L1-like	-1.27	0.0326
LOC100522543	olfactory receptor 140-like	-1.27	0.0381
RPL27A	ribosomal protein L27a	-1.28	0.0322
RPS26	ribosomal protein S26	-1.28	0.0217
LOC100154493	olfactory receptor 2G3-like	-1.28	0.0360
FAM184A	family with sequence similarity 184, member A	-1.29	0.0496
DPH5	diphthamide biosynthesis 5	-1.29	0.0480
ABHD5	abhydrolase domain containing 5	-1.29	0.0373
ZNF641	zinc finger protein 641	-1.29	0.0150

HACL1	2-hydroxyacyl-CoA lyase 1	-1.29	0.0337
SLC44A5	solute carrier family 44, member 5	-1.29	0.0152
SH2D1B	SH2 domain containing 1B	-1.30	0.0482
CLDN2	claudin 2	-1.30	0.0061
TP53TG5	TP53 target 5	-1.31	0.0381
LOC100511607	protein LEG1 homolog	-1.31	0.0078
SFRP1	secreted frizzled-related protein 1	-1.32	0.0167
MIR138	microRNA mir-138	-1.32	0.0324
KIAA1551	KIAA1551 ortholog	-1.34	0.0053
MDM1	Mdm1 nuclear protein	-1.34	0.0186
ZFAND2A	zinc finger, AN1-type domain 2A	-1.34	0.0020
LOC102158467	uncharacterized LOC102158467	-1.34	0.0448
LONRF1	LON peptidase N-terminal domain and ring finger 1	-1.35	0.0373
MOCS1	molybdenum cofactor synthesis 1	-1.37	0.0333
JCHAIN	joining chain of multimeric IgA and IgM	-1.39	0.0064
LOC100524260	zinc finger protein 45	-1.40	0.0381
TRIM25	tripartite motif containing 25	-1.43	0.0028
LOC100525902	calcium/calmodulin-dependent 3,5-cyclic nucleotide phosphodiesterase 1C	-1.45	0.0306
TNFRSF11B	tumor necrosis factor receptor superfamily, member 11b	-1.54	0.0183
CYP2J34	cytochrome P450, family 2, subfamily J, polypeptide 34	-1.61	0.0334
LOC102166306	alpha-fetoprotein-like	-1.71	0.0076
CISH	cytokine inducible SH2-containing protein	-2.50	0.0188

Table S2 qPCR validation of microarray data for selected differentially expressed transcripts

	Fold change		*p*-value
	Microarray	qPCR	qPCR
ASPA	1.70	1.35	0.115
CXCL9	1.69	1.37	0.342
ID2	1.64	1.80	0.279
PQLC2	1.27	1.30	0.138
JUN	1.25	1.23	0.290
GPR18	1.24	2.35	0.416
GMPPB	-1.22	-2.07	0.105
S100A	-1.26	-1.20	0.615
ABHD5	-1.29	-2.17	0.012
DPH5	-1.29	-1.45	0.222
HACL1	-1.29	-1.80	0.024
MOCS	-1.37	-2.03	0.036
JCHAIN	-1.39	-1.67	0.192
TRIM25	-1.43	-1.41	0.378

Microarray and qPCR fold changes were calculated from $n = 6$ animals/group.

Table S3 Characteristics of gene-specific primers used for qPCR analysis

Gene symbol	Primer sequence (forward, reverse)	NCBI GenBank accession no.	Product size (bp)
Reference genes			
ACTB	GACATCCGCAAGGACCTCTA	XM_003124280	205
	ACATCTGCTGGAAGGTGGAC		
ATP5G1	CAGTCACCTTGAGCCGGGCGA	NM_001025218	94
	TAGCGCCCCGGTGGTTTGC		
RPS9	GTCGCAAGACTTATGTGACC	XM_003356050	325
	AGCTTAAAGACCTGGGTCTG		
Target genes			
ASPA	CAGCCTCAAGGAGTTCTGCG	NM_001123077	232
	CCCCAGGGTGCAATGGTTTC		
CXCL9	GCAGTGTTGCCTTGCTTTTGG	NM_001114289	104
	TTGGCTGGTGTTGATGCAGG		
ID2	GCACCCCACTATTGTCAGCC	NM_001037965	240
	AGAGCCTGTGGATTTGTTGCTG		
PQLC2	CTGCAATCTCATCGGCTCCTTC	NM_001243851	181
	CGTCATGCCCACGATGAACAAG		
JUN	ATGACCCTGAACCTGGCTGAC	NM_213880	188
	GGGCATAGGAACTGGGTAGGG		
GPR18	TGACACAAGCGACTCAAAGACG	XM_003131059	153
	GGCCAGGGTGAGTTTGGTTG		
GMPPB	CGGTTCCGGAGCCTCGAATC	NM_001244541	232
	TGAGGGAGTGGGCTTTGTGG		

S100A	AAGTACTCAGGCAAGGAGGGTG	NM_001252605	156
	CTCGTTGTCCCTGTTGCTGTC		
ABHD5	ACACAACCTGGGTGGGTTCC	NM_001012407	114
	TCAGCAAGGTCTGGTCGCTC		
DPH5	TGAACCAAGCAGCACAGCAG	NM_001243679	118
	TCCTCAGCTCCAACTCTGGC		
HACL1	GGAGTCGGTTTGGGATTTGCC	NM_001206454	223
	CTGGAGGGGCCATATCCTTCC		
MOCS	TCAAGGTCTGCCTCTTCGGG	XM_001928708	236
	GATGCTCGGAATGGCTGGTG		
JCHAIN	AGCACCGTCATTTGCACAGTC	XM_003356961	245
	ACTAGGGTCTTCAGCAGAGCG		
TRIM25	TCGAGACTTCTTCTGCCCTG	XM_005656971	163
	CGATGCACCGTTGATCTGAC		
